# Bcl-2^*high*^ mantle cell lymphoma cells are sensitized to acadesine with ABT-199

**DOI:** 10.18632/oncotarget.4230

**Published:** 2015-05-22

**Authors:** Arnau Montraveta, Sílvia Xargay-Torrent, Laia Rosich, Mònica López-Guerra, Jocabed Roldán, Vanina Rodríguez, Eriong Lee-Vergés, Mercè de Frías, Clara Campàs, Elias Campo, Gaël Roué, Dolors Colomer

**Affiliations:** ^1^ Experimental Therapeutics in Lymphoid Malignancies Group, Institut d'Investigacions Biomèdiques August Pi i Sunyer (IDIBAPS), Barcelona, Spain; ^2^ Unitat d'Hematopatologia, Hospital Clinic, IDIBAPS, Barcelona, Spain; ^3^ Advancell-Advanced In Vitro Cell Technologies S.A., Barcelona, Spain

**Keywords:** acadesine (AICAR), mantle cell lymphoma, Mcl-1, ABT-199, Bcl-2

## Abstract

Acadesine is a nucleoside analogue with known activity against B-cell malignancies. Herein, we showed that in mantle cell lymphoma (MCL) cells acadesine induced caspase-dependent apoptosis through turning on the mitochondrial apoptotic machinery. At the molecular level, the compound triggered the activation of the AMPK pathway, consequently modulating known downstream targets, such as mTOR and the cell motility-related vasodilator-stimulated phosphoprotein (VASP). VASP phosphorylation by acadesine was concomitant with a blockade of CXCL12-induced migration. The inhibition of the mTOR cascade by acadesine, committed MCL cells to enter in apoptosis by a translational downregulation of the antiapoptotic Mcl-1 protein. In contrast, Bcl-2 protein levels were unaffected by acadesine and MCL samples expressing high levels of Bcl-2 tended to have a reduced response to the drug. Targeting Bcl-2 with the selective BH3-mimetic agent ABT-199 sensitized Bcl-2 *high* MCL cells to acadesine. This effect was validated *in vivo*, where the combination of both agents displayed a more marked inhibition of tumor outgrowth than each drug alone. These findings support the notions that antiapoptotic proteins of the Bcl-2 family regulate MCL cell sensitivity to acadesine and that the combination of this agent with Bcl-2 inhibitors might be an interesting therapeutic option to treat MCL patients.

## INTRODUCTION

Mantle cell lymphoma (MCL) is an aggressive lymphoid neoplasia characterized by an abnormal proliferation of mature B lymphocytes. It is genetically characterized by the presence of the t(11;14)(q13;q32) translocation causing cyclin D1 overexpression. Additionally, several secondary genetic events contribute to the development and aggressiveness of the disease. Clinically, the prognosis of MCL is generally poor, being among the worst of all B-cell lymphomas [1]. Current standard therapy includes intensive chemoimmunotherapy followed by autologous transplant or rituximab maintenance depending on the age and the fitness of the patient. Although these strategies have high initial response rates, most patients relapse and die from disease-related complications [2]. Within this background, new efforts in the identification of crucial biologic pathways involved in cell survival and proliferation are leading to the development of rational strategies targeting for instance, the proteasome, the phosphoinositide 3-kinase (PI3K)-Akt-mammalian target of rapamycin (mTOR), the B-cell receptor signaling pathway, and the antiapoptotic Bcl-2 family of proteins [2, 3].

In cancer cells, adenosine monophosphate-activated protein kinase (AMPK) signaling pathway is commonly deregulated. AMPK is a highly conserved heterotrimeric serine/threonine kinase that contributes to the control of cell growth, proliferation and autophagy through the downregulation of mTOR activity. AMPK acts as an energy sensor that is activated in response to numerous stress factors, such as hypoxia or glucose deprivation, linking cellular metabolism and energy status to prosurvival signaling pathways [4, 5]. Considering the integral role of this pathway, targeting AMPK might represent a relevant therapeutic strategy for cancer treatment [6–8]. In this context, acadesine (5-aminoimidazole-4-carboxamide-1-D-ribofuranoside, AICA-riboside or AICAR), a cell-permeable nucleoside analogue, is metabolically converted by the cells to AICA ribotide (ZMP), which acts as an AMP mimetic and triggers the AMPK activation [7]. Acadesine has been reported to mimic a low energy state and mediate anticancer effects by different mechanisms, which may depend on the bioenergetic status of the cells [4, 9, 10]. Recent evidences in certain cancer models indicated that acadesine might also inhibit proliferation, independently of the AMPK pathway [11, 12]. In several hematologic malignancies, acadesine has been shown to induce selective apoptosis [13–17] and/or inhibition of proliferation and cell cycle arrest [18, 19]. Furthermore, due to the promising *in vitro* activity of acadesine in CLL cells [13, 14], a phase I/II clinical trial was conducted in relapsed/refractory CLL patients with an acceptable safety profile, and showing that the compound might be effective for the treatment of these patients [20]. Regarding MCL, we previously reported that acadesine was cytotoxic for MCL cells alone or in combination with rituximab [16]. However, the responses among the MCL samples were heterogeneous and the molecular mechanisms implicated in acadesine response were not fully characterized. In this manuscript, we provide insight on the signaling pathways implicated in the activity of the compound in MCL cells and explore a rational combination with ABT-199 to overcome acadesine resistance in MCL.

## RESULTS

### Acadesine induces apoptosis by a caspase-dependent mechanism and activates AMPK

**Figure 1:**
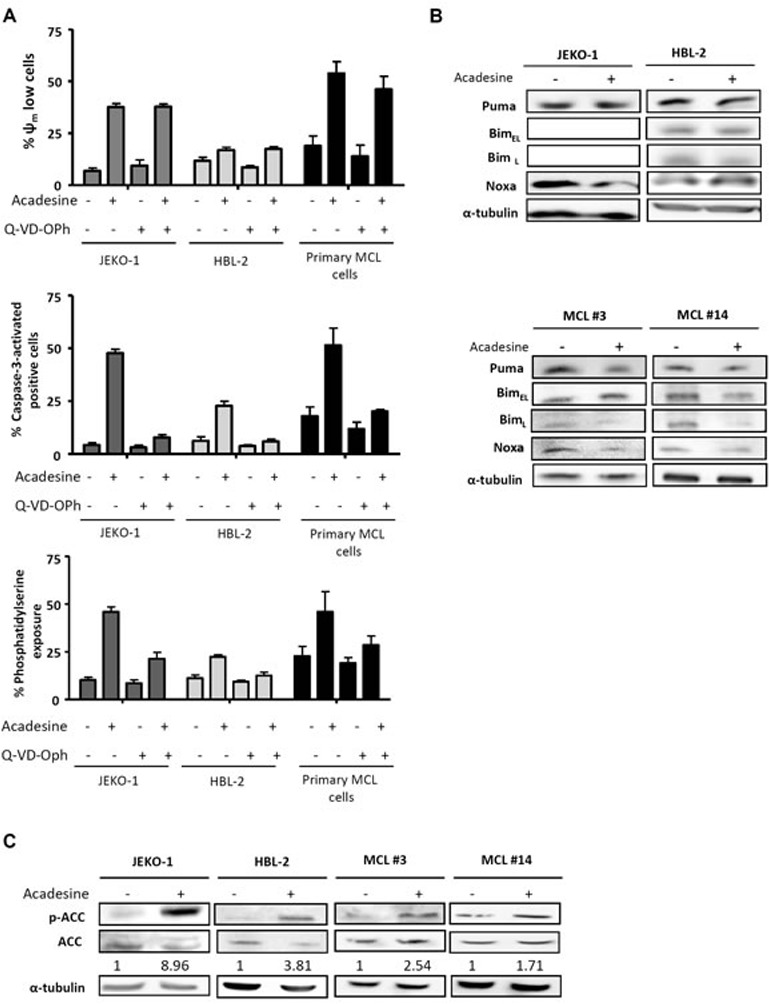
Acadesine induces apoptosis and activates AMPK **A.** JEKO-1, HBL-2 and 3 primary MCL samples were preincubated for 1 hour with 10 μM of the pan caspase inhibitor Q-VD-OPh and followed by a 24-hour exposure to acadesine 2 mM. Mitochondrial membrane potential (Ψ_m_), caspase-3 activation and phosphatidylserine exposure were evaluated by flow cytometry as detailed in “Methods”. **B.** MCL lines (JEKO-1 and HBL-2) and two representative primary MCL samples were cultured with acadesine 2 mM for 6 hours and protein levels of Bim, Puma and Noxa were determined by western blot. α-tubulin was used as loading control. **C.** MCL lines (JEKO-1 and HBL-2) and two MCL primary samples were cultured with acadesine 2 mM for 6 hours. Phosphorylated and total levels of ACC were assessed by western blot using α-tubulin as loading control. The ratio between the phosphorylated and unphosphorylated form was showed.

We previously reported that acadesine was able to induce cytotoxicity in MCL cell lines and primary MCL samples, although some differences in sensitivity were observed among them [16]. With the aim to provide further evidence on the cell death mechanism triggered by the drug in these cells, we analyzed several apoptotic hallmarks. JEKO-1 and HBL-2 cell lines, with different sensitivity to the compound according to our previous results [16], and 3 primary MCL samples were incubated with acadesine (2 mM) for 24 hours and mitochondrial depolarization, caspase-3 activation and phosphatidylserine exposure were analyzed by flow cytometry. In all the samples studied, although at different magnitude, acadesine concomitantly decreased the mitochondrial membrane potential, activated the caspase-3 and increased the phospatidylserine exposure (Figure 1A). On the contrary, when the caspase inhibitor Q-VD-OPh was added, cells were rescued from caspase-3 activation and phosphatidylserine exposure but not from the loss of the mitochondrial membrane potential, indicating that the apoptosis induced by the nucleoside analogue was caspase-mediated (Figure 1A).

Given that in CLL cells acadesine-induced apoptosis has been reported to be mediated by the up-regulation of the proapoptotic BH3-only proteins Bim, Noxa and Puma [15], we examined the levels of these proteins in our model. MCL cell lines and primary MCL cells were incubated with acadesine (2 mM) for 6 hours and BH3-only proteins were analyzed by western blot. As shown in Figure 1B, no upregulation of any of these proteins in the samples studied was detected, suggesting a different mechanism of apoptosis induction in MCL cells. As previously reported, Bim expression was not detected in JEKO-1 cells due to a homozygous deletion at *BIM* locus [21].

Next, we verified whether acadesine was efficiently activating AMPK in MCL cells, as seen in the majority of cell types, including CLL [14]. For this purpose, we assessed the levels of phosphorylation of the AMPK substrate, acetyl-CoA carboxylase (ACC), which is phosphorylated upon AMPK activation [15]. Indeed, as shown in Figure 1C, a 6-hour incubation with acadesine induced ACC phosphorylation in all MCL samples, indicating that acadesine activated the AMPK pathway.

### Acadesine induces VASP phosphorylation concomitantly to an inhibition of CXCL12-induced chemotaxis and cytoskeleton organization

AMPK has been reported to regulate the phosphorylation of the actin regulatory protein vasodilator-stimulated phosphoprotein (VASP) [22]. VASP phosphorylation results in inhibition of actin polymerization, cell adhesion and migration [22, 23]. Gene expression profile studies from our group suggested a potential role of acadesine in reducing the migration of MCL cell lines [16]. In this context, we sought to explore whether the inhibition of migration by acadesine could be related to VASP phosphorylation.

**Figure 2:**
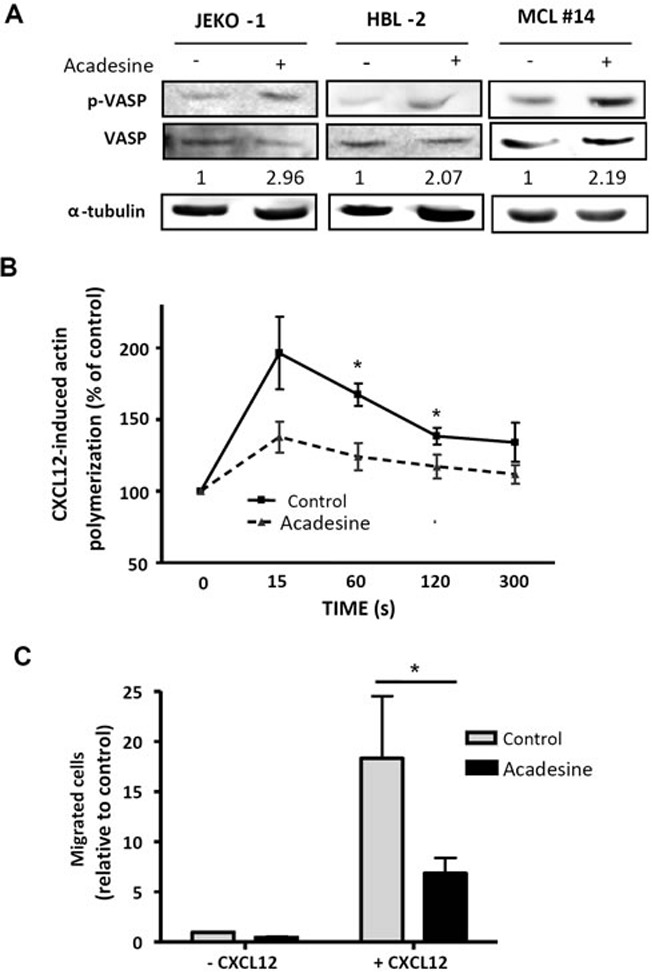
Acadesine phosphorylates VASP and inhibits CXCL12-induced migration **A.** MCL lines (JEKO-1 and HBL-2) and one MCL primary sample were treated with acadesine 2 mM for 6 hours. Phosphorylated and total levels of VASP were analyzed by western blot using α-tubulin as loading control. The ratio between the phosphorylated and unphosphorylated form was showed. **B.** Actin polymerization of 6 MCL primary samples exposed to acadesine (2 mM) was quantified by flow cytometry at the indicated times after CXCL12 stimulation (200 ng/ml). Values were referred to the corresponding unstimulated sample. Mean ± SEM is represented. *P < 0.05. **C.** Chemotaxis toward CXCL12 was performed in 6 MCL primary samples after a 3-hour incubation with acadesine 2 mM as detailed in “Methods”. Bars represent the chemotaxis of the viable cells referred to the control cells without CXCL12. Mean ± SEM is represented. *P < 0.05.

First, we analyzed VASP phosphorylation levels after acadesine (2 mM) exposure for 6 hours. JEKO-1, HBL-2 as well as primary MCL cells showed an increase in phosphorylation levels of VASP after acadesine treatment (Figure 2A). In parallel, we performed actin polymerization assays in the presence of CXCL12, to study if the drug was also able to block this phenomenon. CXCL12 stimulation increased actin polymerization in MCL primary samples that peaked at 15 seconds. A significant inhibition of this process was observed with acadesine incubation at 60 and 120 seconds (*P < 0.05; Figure 2B).

Finally, we performed chemotaxis assays towards CXCL12 stimulus to confirm acadesine effects on cell migration in a set of 6 MCL primary samples. As shown in Figure 2C, acadesine (2 mM) significantly blocked the migration induced by stimulation with CXCL12 (*P < 0.05).

### Acadesine downregulates Mcl-1 through the AMPK/mTOR pathway

**Figure 3:**
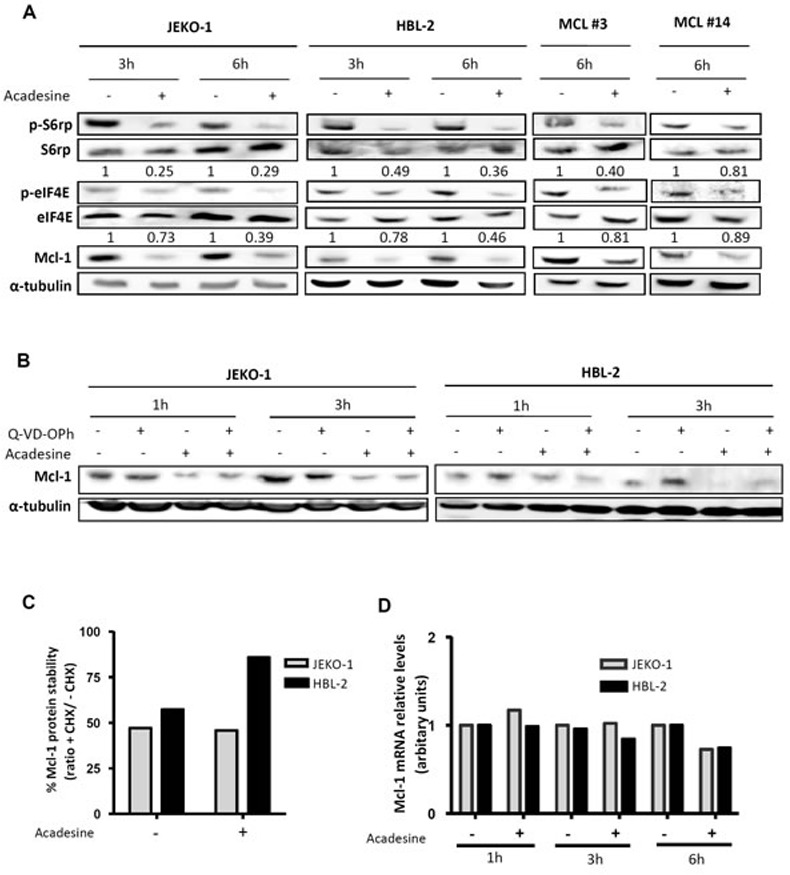
Acadesine downregulates Mcl-1 through the mTOR pathway **A.** MCL lines (JEKO-1 and HBL-2) and two primary MCL samples were incubated with acadesine 2 mM for the indicated times. Phosphorylation of kinases of the AMPK/mTOR pathway and Mcl-1 levels were determined by western blot using α-tubulin as a loading control. The ratio between the phosphorylated and unphosphorylated form was showed. **B.** JEKO-1 and HBL-2 cells were cultured with acadesine 2 mM after a 1-hour incubation with Q-VD-OPh for the indicated times. Mcl-1 levels were detected by western blot using α-tubulin as loading control. **C.** MCL cells (JEKO-1 and HBL-2) were treated for 1 hour with cycloheximide prior to incubation with acadesine 2 mM for 4 hours. Relative levels of Mcl-1 were quantified by densitometry using α-tubulin as a loading control. Bars represent the protein stability considering the ratio with/without cycloheximide. CHX, cycloheximide. **D.** Relative Mcl-1 mRNA levels were quantified in JEKO-1 and HBL-2 after acadesine (2 mM) exposure for the indicated times by qRT-PCR taking as a reference the corresponding untreated condition.

mTOR is a well-known AMPK target that has been involved in MCL pathogenesis [24]. To study the modulation of this pathway by acadesine, we first analyzed the phosphorylation status of several mTOR-related kinases after incubating MCL cells with acadesine (2 mM). Western blot analysis revealed that short-time incubation with acadesine was enough to inhibit the mTOR translational activity, as shown by a decrease in the phosphorylation levels of the mTOR downstream kinase S6 ribosomal protein (p-S6rp) and the eukaryotic translation initiation factor 4E (p-eIF4E) both in MCL cell lines and primary MCL samples (Figure 3A). Simultaneously to mTOR inhibition, we found an important reduction in the levels of the antiapoptotic protein Mcl-1 (Figure 3A). Given that Mcl-1 is regulated by mTOR at translational level [25], we hypothesized that acadesine could decrease Mcl-1 levels by affecting the AMPK/mTOR axis. To confirm that acadesine was interfering with Mcl-1 translation, we then wanted to discard other possible mechanisms explaining its modification. We incubated two MCL cell lines with acadesine and the pan-caspase inhibitor Q-VD-OPh to analyze if Mcl-1 was degraded by caspases. As seen in Figure 3B, acadesine reduced Mcl-1 levels even when cells were treated with the caspase inhibitor, indicating that acadesine-induced Mcl-1 downregulation was not caspase-mediated. We next wanted to examine if acadesine was affecting Mcl-1 stability. MCL cell lines were incubated with acadesine before adding the protein synthesis inhibitor cycloheximide. Cycloheximide addition did not enhance the decrease of Mcl-1 protein levels upon acadesine exposure, indicating that when translation was blocked, acadesine did not increase Mcl-1 elimination (Figure 3C). Finally, we monitored *MCL1* mRNA levels by qRT-PCR to analyze a possible transcriptional regulation of *MCL1* by acadesine treatment. Figure 3D shows that no changes in the expression levels of *MCL1* were detected in JEKO-1 and HBL-2 cell lines after acadesine treatment in any incubation time. Altogether these results suggested that acadesine inhibits the Mcl-1 translation by affecting the AMPK/mTOR pathway.

### Bcl-2 expression confers resistance to acadesine

**Figure 4:**
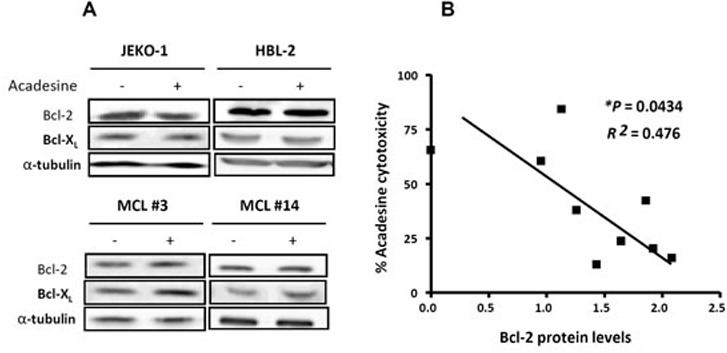
Bcl-2 expression inversely correlates with acadesine sensitivity **A.** MCL cell lines (JEKO-1 and HBL-2) and MCL primary samples were treated with acadesine 2 mM for 6 hours. Protein levels of Bcl-2 and Bcl-X_L_ were detected by western blot using α-tubulin as a loading control. **B.** Correlation between Bcl-2 protein levels assessed by western blot and acadesine cytotoxicity in MCL cell lines (*n* = 9). Relative Bcl-2 levels were quantified by densitometry using α-tubulin as a loading control. Cytotoxic effect was determined after a 24-hour incubation with acadesine 2 mM and referred to the corresponding untreated control.

Because Mcl-1 was downregulated at the same degree in all the samples, irrespective of the cytotoxic response to acadesine, other mechanisms explaining differences in acadesine-induced cytotoxicity were explored. In addition to Mcl-1, the antiapoptotic proteins Bcl-2 and Bcl-X_L_ also play a role in the control of the survival of MCL cells [26]. Therefore, we investigated if the levels of these two proteins were altered after acadesine treatment. As shown in Figure 4A, acadesine treatment did not induce substantial changes in Bcl-2 and Bcl-X_L_ protein levels after 6-hour incubation in MCL cell lines and primary MCL samples. Next, we evaluated putative differences regarding the basal level of these two antiapoptotic proteins in correlation with acadesine sensitivity. For this aim, we quantified by western blot the basal protein levels of Bcl-2 and Bcl-X_L_ in a set of 9 MCL cell lines and analyzed the correlation with the corresponding cytotoxic effect of acadesine (2 mM) at 24 hours of exposure (Table 1). Interestingly, we observed that Bcl-2 protein levels inversely correlated with response to acadesine (*P < 0.05), thus in Bcl-2*^high^* cell lines (HBL-2 and GRANTA-519) acadesine was less effective (Figure 4B). In contrast, no significant association was observed between Bcl-X_L_ protein levels and acadesine sensitivity (data not shown). Since significant association was found between Bcl-2 mRNA and protein levels (**P < 0.01), the Bcl-2 correlation with sensitivity to acadesine was confirmed at gene expression level by RT-PCR in MCL cell lines (*n* = 9) together with primary MCL cases (*n* = 11) (*P < 0.05; Table 1–2).

**Table 1:** Basal mRNA and protein relative levels of antiapoptotic factors in MCL cells

MCL cell lines	Acadesine sensitivity % Cytotoxicity 2 mM	^a^ Protein levels		^b^ mRNA levels	
		Bcl-2	Bcl-XL	*BCL2*	*BCLXL*
GRANTA-519	21.61	1.53	1.02	13.68	1.80
HBL-2	19.81	1.66	0.54	17.23	1.19
JEKO-1	60.43	0.76	0.98	5.94	1.29
JVM-2	37.80	1.00	1.00	1.00	1.00
MAVER	48.19	1.48	1.75	10.99	2.54
MINO	17.71	1.14	1.85	5.96	0.87
REC-1	84.29	0.90	2.04	3.89	1.01
UPN-1	65.45	0.00	1.40	0.00	1.05
Z-138	23.67	1.31	1.93	4.58	2.67

a Protein expression was quantified by densitometry

b mRNA levels were quantified by qRT-PCR

**Table 2:** Biological characteristics and basal mRNA relative levels of antiapoptotic factors in MCL primary samples

MCL primary samples	Gender	Age	Disease status	Previous treatment	% tumoral cell	TP53 status	% acadesine Cytotoxicity 2 mM	^b^ mRNA levels	
								*BCL2*	*BCLXL*
# 1	M	77	D	No	83	wt	^a^ 35.75	2.46	0.78
# 2	M	86	D	No	85	wt	^a^ 19.22	19.96	0.42
# 3	F	78	R	Yes	97	wt	^a^ 90.50	2.79	1.81
# 4	M	83	P	No	89	wt	^a^ 25.71	5.57	1.02
# 5	M	79	D	No	91	del/wt	^a^ 88.80	2.14	0.37
# 6	M	79	D	No	86	del/mut	^a^ 88.71	-	-
# 7	M	79	D	No	86	wt	^a^ 13.01	-	-
# 8	M	57	D	No	80	mut	^a^ 13.25	5.71	3.88
# 9	F	87	D	No	77	mut	^a^ 35.51	-	-
# 10	M	66	D	No	84	del/wt	^a^ 77.30	-	-
# 11	F	63	P	No	77	wt	^a^ 54.30	3.49	0.49
# 12	M	68	R	Yes	76	wt	^a^ 69.21	-	-
# 13	M	81	D	No	97	del/^c^	^a^ 45.48	-	-
# 14	M	59	D	No	94	wt	^a^ 38.81	2.14	0.56
# 15	M	68	D	No	92	UPD/^c^	^a^ 29.35	1.24	0.47
# 16	M	89	R	Yes	85	wt	36.75	4.64	0.15
# 17	M	62	D	No	90	del/mut	54.12	2.38	3.19

Abbreviations: D, diagnosis; R, relapse; P, progression; wt, wild type; del, deletion; mut, mutation; UPD, uniparental disomy

a Data from [16]

b mRNA levels were quantified by qRT-PCR

c Mutations not analyzed

### Targeting Bcl-2 sensitizes Bcl-2^*high*^ MCL cells to acadesine

**Figure 5:**
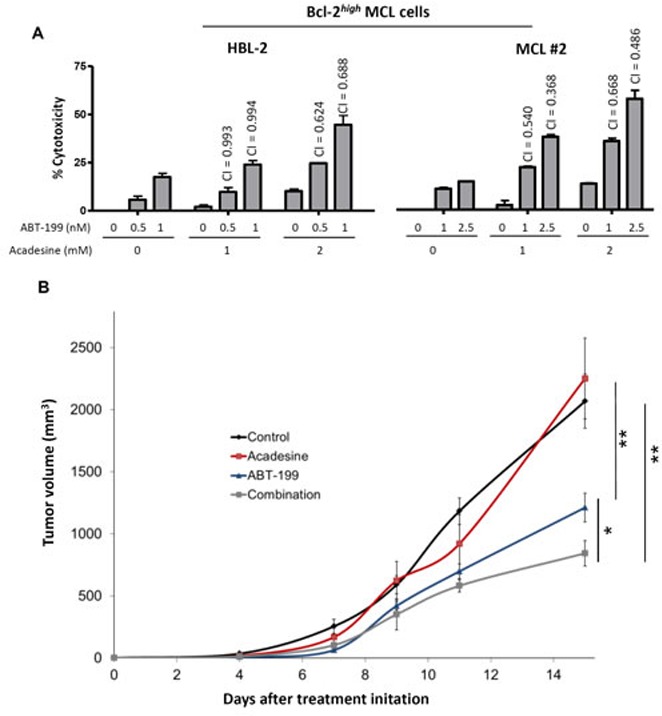
Bcl-2 targeting overcomes acadesine resistance **A.** HBL-2 cell line and a representative Bcl-2*^high^* MCL primary sample were exposed during 24 hours to the indicated doses of acadesine and ABT-199. Graph shows the relative cytotoxicity referred to the untreated samples. CI values are indicated above the bars. **B.** SCID mice were inoculated subcutaneously with HBL-2 cells and treated with acadesine (400 mg/kg 5 days a week), ABT-199 (15 mg/kg weekly) or both drugs. Tumor growth is represented as the mean ± SEM (*n* = 4 mice per group) as detailed in “Methods” (*P < 0.05, **P < 0.01).

As we observed that high levels of Bcl-2 conferred resistance to acadesine, we postulated that targeting this antiapoptotic protein could sensitize Bcl-2*^high^* MCL cells to acadesine. To prove our hypothesis, we treated Bcl-2*^high^* MCL cells simultaneously with acadesine and the BH3-mimetic ABT-199, which selectively inhibits Bcl-2 [27]. First, we tested the combination of both drugs in the representative Bcl-2*^high^* cell line HBL-2 (Figure 5A). Low doses of ABT-199 were able to sensitize this MCL cell line to acadesine, with the combination reaching a similar profile of sensitivity to the one observed for acadesine alone in a Bcl-2*^low^* cell line (JEKO-1; Figure 1A). The combination index (CI) values indicated strong synergism between the two compounds especially when acadesine was used at the highest dose (2 mM). Importantly, these findings were validated in a Bcl-2*^high^* MCL primary case where the combination was also found to be highly synergistic (Figure 5A) as well as in the Bcl-2*^high^* cell line GRANTA-519 (data not shown).

The efficacy of the combination acadesine-ABT-199 was validated *in vivo* in a mouse xenograft MCL model. The representative Bcl-2*^high^* cell line HBL-2 was subcutaneously inoculated to SCID mice. The tumor growth was periodically examined and after 5 days of inoculation, mice were given the corresponding vehicle, acadesine (400 mg/kg 5 days a week) and/or ABT-199 (15 mg/kg weekly). Figure 5B showed that after 15 days of treatment acadesine alone was ineffective in reducing tumor growth. On the contrary, ABT-199 displayed a notable activity in monotherapy with a reduction of 42% in tumor size (**P < 0.01), whereas the regimen of acadesine plus ABT-199 achieved a 59% of tumor regression (*P < 0.05, **P < 0.01; Figure 5B).

## DISCUSSION

Acadesine has been shown to induce antitumor activity in several cancer models, by affecting apoptosis [28, 29], autophagy [30] or cell proliferation [10, 12, 17–19, 31]. In B-cell neoplasms the nucleoside analogue has been extensively studied both *in vitro* [13, 14] and in clinical trials [20]. Recently, we reported that acadesine had an heterogeneous cytotoxic effect in a wide range of MCL cell lines and primary samples alone or in combination with rituximab [16]. In the present manuscript we provide further insight on the molecular mechanism of action of acadesine in MCL and explore potential strategies to overcome acadesine-resistance.

We confirmed the activation of the AMPK pathway by acadesine in MCL cells, as reported in other models [6, 7, 10, 11, 14, 29]. AMPK has been found to be inactivated in lymphoma [32, 33] and has specific roles in many processes that critically impact tumor progression [34]. One of such functions is to regulate cell migration through VASP phosphorylation [22, 35]. VASP is an actin binding phosphoprotein implicated in the control of actin cytoskeleton elongation and cell migration [36–39], whose phosphorylation at Ser239 impairs actin assembly [22, 36, 37]. Consistently, we observed that in MCL cells, acadesine triggered VASP phosphorylation at Ser239, as well as blockade of actin polymerization and migration in MCL cells. Chemokine signaling and adhesion processes are regulating the trafficking of MCL cells, and their homing and/or retention within tissue microenvironments. Thus, therapeutic approaches disrupting these phenomena are acquiring more relevance in MCL [40]. *In vitro* studies demonstrated that several kinase inhibitors [41–44] as well as the recently approved BTK inhibitor ibrutinib [45] blocked these migratory and microenvironmental survival signals. At the clinical setting, the inhibition of these processes results in a mobilization of tumor cells from the lymphoid tissues to the peripheral blood [45, 46]. In accordance, a similar trend of mobilization was observed in the phase I/II clinical trial conducted with acadesine in relapsed/refractory CLL patients, where an increase in the CLL counts in peripheral blood and a reduction of the lymph node size was observed [20].

It is also well-known that AMPK regulates mTOR signaling [4, 5, 8]. In MCL cells, where the PI3K/Akt/mTOR survival pathway is constitutively activated [24], AMPK activation results in an inhibition of the mTOR cascade [47]. Herein, we confirmed that a short incubation with acadesine efficiently inhibited the mTOR translational activity in MCL cells, by dephosphorylation of the mTOR downstream factors S6rp and eIF4E. This decrease on phosphorylation was less evident by eIF4E that might be due to the regulation of this protein by other signaling pathways [48]. Furthermore, we demonstrated a critical reduction in the levels of the antiapoptotic protein Mcl-1 as a consequence of a blockade in its translation. Our findings indicated that the downregulation of this protein is neither transcriptional nor through enhanced degradation, highlighting the direct connection between mTOR inhibition and Mcl-1 downregulation. Supporting this, other compounds that target mTOR pathway were also described to inhibit Mcl-1 at a translational level in lymphomas [44, 49, 50].

Our results showed that acadesine ultimately activated the mitochondrial apoptotic pathway and caused caspase-dependent cell death in MCL, in accordance to what has been observed in CLL [14]. Activation of apoptosis might be consequence of either upregulation of the proapoptotic members of the Bcl-2 family or downregulation of the antiapoptotic ones. [51]. In CLL, acadesine-induced apoptosis is promoted by the BH3-only proteins Bim, Puma or Noxa [15]. However, we did not find upregulation of these factors in MCL cells, suggesting a distinct mechanism of apoptosis activation. In this context, the reduction of Mcl-1 induced by acadesine exposure might account for MCL entrance in apoptosis, similarly to what observed with other drugs [44, 52]. Nevertheless, although a similar reduction of Mcl-1 protein levels was observed among all the MCL cases, a variable degree of cytotoxic response to acadesine was distinguished, suggesting that other mechanisms might influence acadesine-induced cell death. Remarkably, we found that high *BCL2* expression correlated with a reduced response to acadesine in MCL cells. In line with this, B lymphocytes in vav-*BCL2* transgenic mice are highly resistant to acadesine treatment [15] as well as primary follicular lymphoma samples, which constitutively express high levels of Bcl-2 due to the t(14;18) translocation [13].

BH3-mimetic drugs have emerged in order to overcome the mechanisms of apoptosis resistance by targeting Bcl-2 family of proteins. ABT-199 is the most selective BH3 mimetic against Bcl-2, which specifically binds to Bcl-2 but not to Mcl-1 and Bcl-X_L_ [27]. The compound has shown promising clinical activity in early studies [3]. In hematological malignancies, including CLL and MCL, ABT-199 induces apoptosis [53] and its activity was found to directly correlate with Bcl-2 expression in a set of lymphoma cell lines [27]. In this context, we demonstrated that nanomolar doses of ABT-199 are able to sensitize Bcl-2*^high^* MCL cells, to acadesine. This strategy was also validated *in vivo* using a subcutaneous model of the HBL-2 cell line, where the combination of acadesine plus ABT-199 was more potent in reducing tumor growth than each drug alone. Of note, the addition of low doses of ABT-199 to acadesine was much less effective in Bcl-2*^low^* MCL cell lines (data not shown), probably reflecting their minor Bcl-2 dependency. Acadesine might also have a role in resensitizing ABT-199 resistant cells. High levels of Mcl-1 and Bcl-X_L_ have been reported to be a mechanism of both de novo [54] and acquired [55] resistance to ABT-199 in lymphoma cell lines, which is overcome by the addition of mTOR inhibitors [55], and possibly with acadesine. Given that the majority of cancers, including MCL, lack an “Achilles heel”, there is growing evidence that combination therapies can control tumor growth more efficiently [56].

Even though acadesine is a nucleoside analogue, its antitumoral mechanistic effect differs from conventional chemotherapeutic agents in MCL. Collectively, our findings suggest that this drug modulates downstream AMPK targets affecting MCL migration and inhibiting the mTOR pathway. Furthermore, we demonstrated both *in vitro* and *in vivo* that acadesine resistance found in Bcl-2*^high^* MCL samples might be overcome by targeting Bcl-2 with ABT-199. These results indicate that acadesine in combination of BH3-mimetics might represent a promising therapeutic option to treat MCL patients.

## MATERIALS AND METHODS

### MCL cell lines and primary cultures

Nine human MCL cell lines (GRANTA-519, JVM-2, JEKO-1, Z-138, MAVER-1, REC-1, UPN-1, HBL-2 and MINO; Table 1) were cultured with RPMI-1640 or Dulbecco's modified Eagle's medium (DMEM) medium complemented with 10-20% heat-inactivated FBS, 2 mM L-glutamine, 50 μg/ml penicillin/streptomycin (Life Technologies). Cells were grown in a humidified atmosphere at 37C° with 5% carbon dioxide and routinely tested for *Mycoplasma* absence by PCR. Additionally, the genetic identity of all cell lines was periodically verified using the AmpFISTR identifier kit (Life Technologies). Genetic characterization of these MCL cell lines has been previously reported [44]. Primary MCL cells were obtained from peripheral blood samples of MCL patients diagnosed according to the WHO criteria. The ethical approval for this project, including the informed consent of the patients, was granted following the guidelines of the Hospital Clínic Ethics Committee (IRB). Biological characteristics of these cases are shown in Table 1. Tumor cells were isolated by centrifugation on a Ficoll-Hypaque (GE Healthcare) gradient and conserved within the Hematopathology collection registered at the Biobanc from Hospital Clínic-IDIBAPS (R121004-094). Cells were cultured in supplemented RPMI as above.

### Drugs and detection of apoptosis by flow cytometry

Cells were incubated with acadesine (1 and 2 mM; kindly provided by Advancell) and/or ABT-199 (0.5-2.5 nM; Selleck Chemicals) for the times specified. When indicated, cells were preincubated for 1 hour with 10 μM of the pan-caspase inhibitor Q-VD-OPh (Calbiochem). Experiments of protein stability were done by incubating cells for 4 hours with acadesine and then for 1 hour with 100 μM of the protein synthesis inhibitor cycloheximide (Sigma). Cytotoxicity was analyzed by quantification of phosphatidylserine exposure through double staining with Annexin V-fluorescein isothiocyanate (FITC) and propidium iodide (PI; Bender Medsystems). Changes in mitochondrial membrane potential (Ψ_m_) and caspase-3 activation were evaluated by staining cells with 20 nM 3,3′-diexyloxacarbocyanine iodide or Cell Event TM Caspase 3/7 (Life Technologies), respectively. Labeled cells were analyzed on an Attune acoustic cytometer (Life Technologies). CI values were calculated after exposure of cells to increasing concentrations of acadesine and ABT-199 using the Calcusyn software v2.0 (Biosoft). The interaction between drugs was considered synergistic when CI < 1.

### Western blot analysis

Whole-cell protein extracts were obtained by lysing cells with Triton buffer (20 mM Tris-HCL pH 7.6, 150 mM NaCl, 1 mM EDTA, and 1% Triton X-100) supplemented with protease and phosphatase inhibitors (10 μg/mL leupeptin, 10 μg/mL aprotinin, 1 mM phenylmethanesulfonyl fluoride, 5 mM NaF, and 2 mM Na_3_VO_4_; Sigma). Proteins were quantified by using Bio-Rad Protein Assay (Bio-Rad) and separated in 10-15% SDS-PAGE and transferred to an Immobilon-P membrane (Millipore). Membranes were blocked with 2.5% phosphoBlocker Blocking Reagent (Cell Biolabs) in Tris-Buffered Saline (TBS)-Tween 20, and probed with antibodies against: Puma (Abcam), Bim, Noxa (Enzo life sciences), phosphorylated-Ser79 AAC (p-ACC), ACC, phosphorylated-Ser239 VASP (p-VASP), VASP, phosphorylated-Ser235/236 S6rp (p-S6rp), S6rp, phosphorylated-Ser 209 eIF4E (p-eIF4E), eIF4E (Cell Signalling Technology), Mcl-1, Bcl-2 and Bcl-X_L_ (Santa Cruz Biotechnology). Antibody binding was detected using secondary peroxidase-labeled anti-mouse (Sigma) and anti-rabbit (Cell Signaling Technology) antibodies and chemiluminiscence was detected using a mini-LAS4000 Fujifilm device (Fujifilm). Equal protein loading was confirmed by probing membranes with α-tubulin antibody (Sigma). Densitometric quantification was done by using Image Gauge software (Fujifilm).

### Actin polymerization assay

MCL cells (10^7^ cells/mL) were washed twice and serum starved for 1.5 hours in FBS-free RPMI-1640. Acadesine was added for 3 additional hours, and MCL primary cells were diluted to 2 × 10^6^ cells/mL in RPMI-1640 with 0.5% bovine serum albumin (BSA; Sigma). Thereafter, samples were stimulated with 200 ng/mL of CXCL12 (Peprotech) and at the indicated time points, 100 μL of the cell suspension was collected and added to 25 μL of the staining solution [2.5 ng/mL phalloidin-Tetramethyl Rhodamine Isothiocyanate (TRITC), 2.5 mg/mL of L-α-lysophosphatidylcholine (Sigma) and 5% paraformaldehyde (Aname)] for 20 minutes at 37°C. Cells were acquired on an Attune cytometer, and results were plotted relative to the mean fluorescence of the sample before the addition of CXCL12.

### Chemotaxis assay

MCL cells (10^7^ cells/mL) were washed twice and serum-starved for 1.5 hours in FBS-free RPMI. Acadesine 2 mM was added for 3 additional hours, and cells were diluted to 5 × 10^6^ cells/mL with 0.5% BSA in RPMI. One hundred μL (5 × 10^5^ cells) was added to the top chamber of a transwell culture polycarbonate insert with 6.5 mm diameter and 5 μm of pore size (Corning). Inserts had been previously transferred to wells containing 600 μL of RPMI with 0.5% BSA and 200 ng/mL of human recombinant CXCL12. After 3 hours of incubation at 37°C, 100 μL was collected in triplicate from each lower chamber and counted in an Attune cytometer for 12 seconds under constant flow rate. Migration levels of viable cells are given taking the untreated cells without CXCL12 as reference.

### mRNA quantification by real-time qRT-PCR

Total RNA was extracted using Trizol^®^ method (Life Technologies) according to manufacturer's instructions. One microgram of RNA was retrotranscribed to cDNA with M-MLV reverse transcriptase (Life Technologies) and random hexamer primers (Roche). *MCL1*, *BCL2* and *BCLXL* expression was analyzed in duplicate using predesigned Assay-On-Demand probes (Life Technologies) on a StepOne device (Life Technologies). The relative expression of each gene was quantified by the comparative cycle threshold (Ct) method (ΔΔCt) by using *BACTIN* as endogenous control. Expression levels are given in arbitrary units, taking as a reference either the control sample (untreated cells) or the JVM-2 cell line expression samples for *BCL2 and BCLXL*.

### Xenograft model

Six-week-old CB17-severe combined immunodeficient female mice (SCID; Janvier Labs) were inoculated subcutaneously into the right flank with 1:1 of 8 × 10^6^ HBL-2 cells in PBS and Matrigel^®^ basement membrane matrix (Becton Dickinson), according to a protocol approved by the animal testing ethical committee of the University of Barcelona (Barcelona). Five days after the cell injection, mice were randomly assigned into 4 cohorts of 4 each. Acadesine (400 mg/kg) or vehicle were administered by intraperitoneal injection 5 days/week. ABT-199 (15 mg/kg) diluted in 60% phosal 50 PG (Lipoid), 30% poliethylenglicol and 10% ethanol (Sigma) or vehicle were given orally at once a week. The shortest and longest diameters of the tumor were measured with external calipers twice a week and tumor volumes were estimated using the following formula: (the shortest diameter)^2^ x (the longest diameter) x 0.5. Mice were sacrificed after 15 days of the treatment according to the institutional guidelines.

### Statistical analysis

Data were depicted as the mean ± SEM of 3 independent experiments for cell lines or the mean ± SEM for the MCL primary cases. All statistical analyses were done by using GraphPad Prism 4.0 software (GraphPad Software). Comparison of means between 2 groups of samples was evaluated by nonparametric Mann-Whitney test or Wilcoxon paired test. Correlations between two continuous variables were done by a Sperman test. Results were considered statistically significant when *P* < 0.05 (*, *P* < 0.05; **, *P* < 0.01).
